# Intrauterine Bigatti Shaver (IBS®) successful placental remnants removal, after caesarean section for a cervical pregnancy with placenta accreta

**DOI:** 10.52054/FVVO.14.1.010

**Published:** 2022-04-04

**Authors:** J Shi, Y Zhang, S Zhang, X Yin, D An, J Zhang, J Cheng, Y Wang, A Zhao, W Di, R Campo, G Bigatti

**Affiliations:** Sino European Life Expert Centre, Renji Hospital, Shanghai Jiao Tong University School of Medicine; Department of Obstetrics and Gynaecology, Renji Hospital, Shanghai Jiao Tong University School of Medicine; Department of Ultrasound, Renji Hospital, Shanghai Jiao Tong University School of Medicine; Department of Radiology, Renji Hospital, Shanghai Jiao Tong University School of Medicine; Life Expert Centre, Leuven, Belgium

**Keywords:** Operative hysteroscopy, placenta accreta, Intrauterine Bigatti Shaver

## Abstract

Placenta accreta located in a caesarean section scar is difficult to remove. The Intrauterine Bigatti Shaver (IBS®) has already been proven to be effective in placental remnant removal. Our case report highlights that the IBS® is also a safe method to remove placental remnants attached to a previous caesarean section scar performed for a cervical pregnancy and associated with placenta accreta.

## Introduction

According to the literature in the case of a cervical pregnancy with a placenta accreta located inside a previous caesarean section scar, early pregnancy resolution is suggested. However, termination of a second trimester pregnancy might cause severe complications such as haemorrhagic shock, infection, bladder injury, partial placental remnants or even hysterectomy. Among these, placental remnants are the most frequent complication reported after induced labour.

Hysteroscopy has confirmed its efficacy in placental remnants removal (Golan et al., 2011; Hooker et al., 2016). Additionally, the Intrauterine Bigatti Shaver (IBS^®^) has been proven to be a safe and effective technique for placental remnants retrieval (Ansari et al., 2018). This case report describes placental remnants located inside a uterine caesarean section scar previously performed for a cervical pregnancy with placenta accreta, completely removed with the IBS^®^.

## Case Presentation

A 31-year-old woman with a previous history of one caesarean section and one dilation and curettage (D&C) was admitted to our hospital with vaginal bleeding at 13+4 weeks of gestation. Transvaginal sonography and magnetic resonance imaging revealed a cervical pregnancy with a foetus (BPD 26 mm) and an active heartbeat.

**Figure 1:**
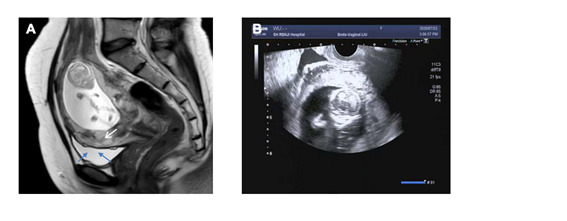
A: MRI showing on the T2W uterine median sagittal plane the placenta completely covering the cervical internal orifice and being accrete to the muscular layer. Part of the placenta breaks through the muscular layer and protrudes into the bladder cavity like a tent (see black arrow). B: Ultrasound showing the presence of a live foetus with placenta previa and its partial uterine implantation.

The internal cervical os was completely covered by the placenta which was found to be adherent to the previous caesarean section scar (Fig. 1). The clinical diagnosis was suggestive for a cervical pregnancy with placenta accreta attached to the previous caesarean section scar.

Due to high risk of massive haemorrhage and uterine rupture the patient decided to terminate the pregnancy. Firstly, drug induction combined with prophylactical uterine artery embolisation (UAE) was attempted. The patient was prescribed mifepristone tablets 25mg twice daily for 2 days and misoprostol 600mcg vaginally but this was not effective. Ethacridine Lactate (100 mg) in 20 mL of 0.9% sodium chloride solution was directly injected into the amniotic fluid with no success. As medical methods of terminating the pregnancy failed, a caesarean delivery was performed. During the operation, the bladder was found to be adherent to the previous caesarean scar. Dissection of the uteri- vesical peritoneal fold was carried out to expose the lower uterine segment, which was oedematous and dark red showing a 10mm dehiscence. The foetus and most of the placenta were removed by means of an oval forceps and additional curettage.

Double layer interrupted sutures were used to stop bleeding and to close the uterine incision. The total amount of intraoperative blood loss was 1200 mL. Three units of packed red blood cells and 600mL of fresh frozen plasma were administered intravenously during the procedure.

Cefradine capsules (250mg 6-hourly) for 3 days and mifepristone tablets (25mg twice daily) for three months were prescribed postoperatively. Histological examination showed chorionic villi and placental tissue infiltrating deeply into the myometrium layer.

**Figure 2:**
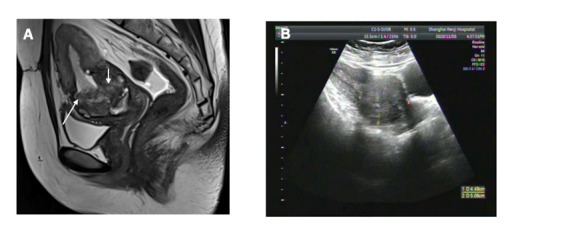
A: MRI showing at T2W sagittal plane a discontinuous lower uterine segment incision (see long white arrow) with the presence of a large mixed soft haemorrhage mass (see short white arrow). B: Ultrasound showing a dilated lower uterine segment with the presence of a heterogeneous mass.

Two months after operation, a transvaginal ultrasound and magnetic resonance imaging showed the presence of a 45 x 51 x 51mm vascularised hypo- and hyper-echogenic tissue attached to the previous caesarean section scar. The uterine cavity was regular with a normal endometrium (Fig. 2). A serum human chorionic gonadotropin (hCG) test was negative.

Diagnostic hysteroscopy was performed to evaluate the uterine cavity as well as the placenta remnants’ volume and vascularisation. Operative hysteroscopy with the Intrauterine Bigatti Shaver (IBS^®^) was performed. The 24Fr. optical system with SA blade of the shaver was used. The rotational speed of the blade was between 2100 to 1500 rpm with a suction of 250/500 mL per minute. The cervical canal was distended by the presence of a 50 x 50 x 50mm dark red tissue mixed with fibrotic placenta and old blood clots densely adherent to the caesarean section scar. The lower segment of the uterus and anterior wall of the cervical canal could not be clearly identified as it was replaced by a very large niche.

**Figure 3:**
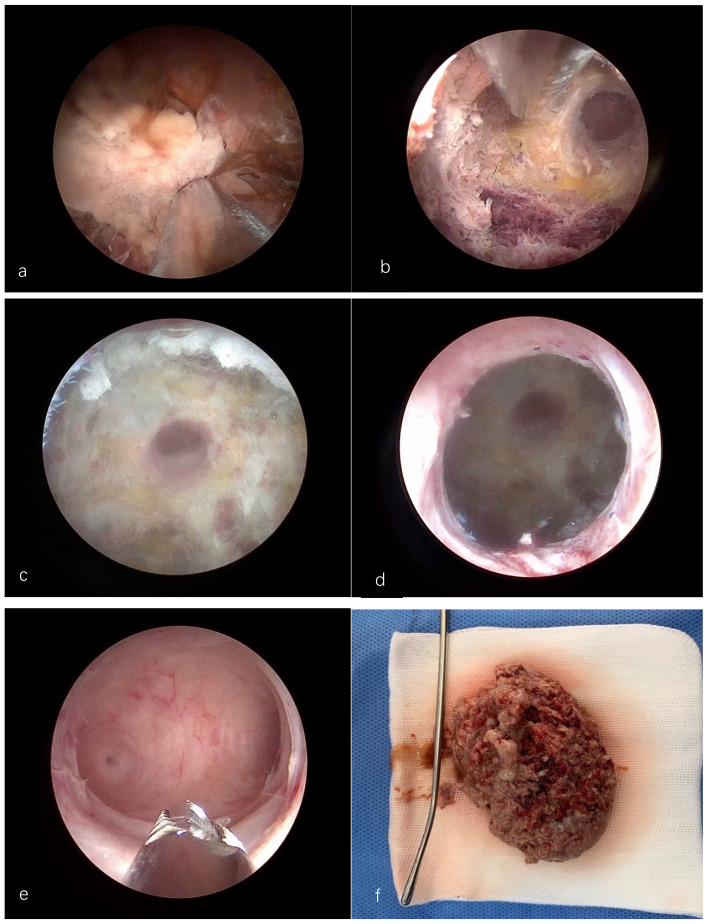
a) Cervical Placental Remnants b) Placental remnants Shaver removal c) Niche with clear caesarean section scar and visible internal uterine orifice d)Post caesarean niche, completely tissue free e) Regular uterine cavity f) Total amount of tissue removed.

Placental remnants were completely removed without bleeding (Fig. 3). The procedure lasted 20 minutes with a fluid deficit of approximately 240mL of normal saline. No intraoperative complication was observed. The histological result confirmed the presence of degenerated villi compatible with placental remnants.

The patient was discharged from hospital the following day with no postoperative complications. At 3 months follow up, the patient didn’t feel any discomfort and experienced regular periods. Written permission of this patient was obtained.

## Discussion

With the increasing number of caesarean sections, complications such as placental remnants, caesarean scar defects, bleeding and uterine rupture are frequently reported. In China the incidence of post caesarean scar defects, due to the high caesarean rate which ranges between 45%-50%, is increasing (Mi and Liu, 2014).

In cases of placenta accreta or increta the removal can be extremely difficult and incomplete. Several medical treatments such as methotrexate and mifepristone as well as ultrasound-guided D&C after bilateral UAE, and even laparoscopic or transabdominal hysterectomy have been proposed (Committee Opinion., 2012; Silver et al., 2015; Wong and Burke, 2012; Lorenz., 2013). Presently operative hysteroscopy aims to be the gold standard procedure for this indication (Golan et al.,2011; Hooker et al., 2016; Ansari et al., 2018).

With the introduction of the Shaver technique (Bigatti.,2011; Bigatti et al.,2012), a new alternative method to the conventional resectoscope has been proposed. According to a recent paper by Ansari et al. (2018) the advantages related to this new technique can be summarised as:

1. Removal of the tissue fragments at the same time of resection
2. Better visualisation
3. Fast and safe procedure with lower complication rate
4. No use of mono or bipolar current with no endometrial heating and therefore, less postoperative adhesion formation.

To increase the success rate of tissue removal systems approach, additional suggestions have been proposed to reduce the intraoperative bleeding problems (Hamerlynck et al., 2013).

These suggestions have been confirmed by Ansari et al. (2018) and consist of waiting at least 2-3 months from the pregnancy date before approaching placental remnants with operative hysteroscopy. Additional indications, arising from the observations of the present case report, consist of performing the procedure in a fast way and starting the placental remnants removal from their upper part before reaching the inflamed endometrial layer. Additionally, the speed reduction of 1500 rotation per minute with an increased suction of the blade to 500 mL/min has significantly reduced the time of the whole procedure. Placental remnants removal should always be performed under 2D transabdominal ultrasound surveillance to guide the surgeon in case of heavy bleeding. Ultrasound will confirm, in case of reduced visibility at hysteroscopy, the right position of the shaver blade inside the uterine cavity. Lastly, 20 IU of Oxytocin in 500 mL of normal saline intravenously should be infused during the whole procedure, to reduce bleeding by means of uterine contraction.

## Conclusion

The success of this case report indicates that the IBS can be considered a valid alternative to all conventional methods used for placental remnants even if they arise from the treatment of a cervical pregnancy with placenta accreta. Additional randomised controlled studies should be performed to confirm the validity of this new technique for placental remnants’ retrieval.
